# Osteoarthritis Pain in Old Mice Aggravates Neuroinflammation and Frailty: The Positive Effect of Morphine Treatment

**DOI:** 10.3390/biomedicines10112847

**Published:** 2022-11-08

**Authors:** Giada Amodeo, Silvia Franchi, Giulia Galimberti, Laura Comi, Simona D’Agnelli, Marco Baciarello, Elena Giovanna Bignami, Paola Sacerdote

**Affiliations:** 1 Dipartimento di Scienze Farmacologiche e Biomolecolari, University of Milan, Via Vanvitelli 32, 20129 Milano, Italy; 2 Anesthesiology, Critical Care and Pain Medicine Division, Department of Medicine and Surgery, University of Parma, Via Gramsci 14, 43126 Parma, Italy

**Keywords:** osteoarthritis pain, neuroinflammation, aging, morphine, frailty, monoiodoacetate, hyperalgesia, allodynia, locomotor activity

## Abstract

Knee osteoarthritis is a common cause of pain and disability in old subjects. Pain may predispose to the development of frailty. Studies on mechanisms underlying pain in osteoarthritis models during aging are lacking. In this work, we used the monosodium iodoacetate model of osteoarthritis in adult (11-week-old) and old (20-month-old) C57BL/6J mice to compare hypersensitivity, locomotion, neuroinflammation, and the effects of morphine treatment. After osteoarthritis induction in adult and old mice, weight-bearing asymmetry, mechanical allodynia, and thermal hyperalgesia similarly developed, while locomotion and frailty were more affected in old than in adult animals. When behavioral deficits were present, the animals were treated for 7 days with morphine. This opioid counteracts the behavioral alterations and the frailty index worsening both in adult and old mice. To address the mechanisms that underlie pain, we evaluated neuroinflammatory markers and proinflammatory cytokine expression in the sciatic nerve, DRGs, and spinal cord. Overexpression of cytokines and glia markers were present in osteoarthritis adult and old mice, but the activation was qualitatively and quantitatively more evident in aged mice. Morphine was able to counteract neuroinflammation in both age groups. We demonstrate that old mice are more vulnerable to pain’s detrimental effects, but prompt treatment is successful at mitigating these effects.

## 1. Introduction

Chronic pain is a health problem worldwide and it is particularly problematic in older adults where it is associated with increased suffering, social isolation, and disability.

Osteoarthritis (OA) of the knee is the most common form of musculoskeletal disease and a frequent cause of chronic pain in the elderly population; indeed, it has been estimated that up to 10% of people over the age of 60 years is affected by some form of OA [1]. Although mechanisms at the basis of the disease are not completely known, it has a multifactorial pathogenesis, in which inflammation, mechanical, and metabolic factors are the causes of progressive joint damage [2,3]. It is a whole joint disease, involving hyaline articular cartilage destruction, inflammation of the synovial membrane, sclerotic changes of subchondral bone, ligaments damages, fibrosis and inflammation of the infrapatellar fat pad, and the formation of bone osteophytes [4]. Finally, the disease is characterized by alterations arising from an imbalance between the repair and destruction of joint tissues [5]. Pain is often present in OA and is the main symptom that drives OA patients to seek medical help [6,7]. Osteoarthritis has inflammatory and neuropathic pain characteristics. Joint cells, such as synoviocytes, inflammatory cells, chondrocytes, as well as infrapatellar fat pads produce chemokines, cytokines, and proteases, which sensitize primary sensory afferents [8,9,10,11]. The increase in nociceptive input from the periphery results in central sensitization in the dorsal horn of the spinal cord where neurons become hyperexcitable [12,13], reducing their thresholds and enhancing their responses. Sensitized neurons expand their receptive fields, spreading hypersensitivity from the knee to adjacent areas, leading to the reduction of the mechanical threshold around the joint, a phenomenon observed in OA patients [14]. Using OA rodent models, we and others described the development of neuroinflammation in dorsal root ganglia (DRGs) and spinal cords that experience the persistence of chronic pain [15,16,17,18,19,20]. Neuroinflammation is characterized by microglia and astrocyte activation and the production of cytokines and chemokines that sensitize neurons. Although age-related changes in nociceptive processes were suggested and despite age being one of the strongest risk factors for OA [21], most studies investigating OA pain and its underlying neuroinflammatory mechanisms use young adult animals. Moreover, in OA-patients, the importance of enhancing physical activity recovery (besides relieving pain) is now recognized. This aspect has been scarcely addressed in OA animal models, which focus mainly on pain outcomes. In elderly populations, effective treatment of OA pain remains an important clinical concern [22]. The efficacious (and prompt) management of OA pain in older adults are really important since the presence of chronic pain predisposes to frailty and dementia and increases the risk of death [22,23]. Opioids are potent analgesics and their efficacy is proven in OA pain, but some unwanted effects have limited their use [22]. On these bases, it is clear that there is a strong need to better study OA pain, its neuroinflammatory mechanisms, and the efficacy of opioids in aging. The monosodium iodoacetate (MIA)-induced OA model is one of the most validated and used in rodents to assess pain and its therapies [15,17,18,20,21]. MIA injection into the intraarticular space (IA) results in functional impairment that resembles human OA [15,17]. MIA animals develop pain-related behaviors, such as thermal hyperalgesia and mechanical allodynia [15,17,24,25,26]. In this study, we address some of the gaps in the OA pain research reported above. We use MIA to induce OA in adult and old mice. Sensory phenotypes, motor activity, muscle strength, and frailty in adult and old healthy and OA mice were evaluated and compared. The impact of OA and age on neuroinflammation in the nerve, DRGs, and spinal cords were assessed by measuring the expression of cytokines and of astrocyte, microglia, and cell damage markers. Moreover, we investigated the effect of a 1-week morphine treatment on pain, locomotor activity, neuroinflammation, and the frailty index to verify the final ratio between the risks and benefits of opioids in aged animals.

## 2. Materials and Methods

### 2.1. Animals

Adult (11 weeks of age, 24 grams of weight, n = 60) and old (20 months of age, 30 grams of weight, n = 60) C57BL/6J male mice (Charles River Laboratories, Calco, Italy) were used. Mice were housed as three per cage (type II—26 cm × 20 cm × 14 cm) with bedding and nesting material, in an enriched environment, with 12-h dark/light cycles at 22 ± 1 °C RT, a humidity of 55 ± 10%, and food (standard pellet) and water ad libitum. Old mice used in the studies arrived at our animal facility at the age of 6 months and were housed until they were 20 months old, reaching old age [27]. During the housing, animals were subjected to periodic veterinary checks to assess their health. No animals at the end of the aging period were excluded from the study. Adult animals were ordered two weeks before experiments. One week before the OA induction, adult and old animals were handled for a few minutes/days. The ARRIVE guidelines were adhered to in the experiments. Animal experimental procedures were approved by the Animal Care and Use Committee of the Italian Ministry of Health (Authorization 180/2020 to PS) and complied with the International Association for the Study of Pain and the European Community (E.C.L 358/118/12/86). The suffering and the number of animals used were reduced accordingly with the 3R principles. Correct strategies to minimize potential confounders were applied. Tests with adult and old mice were performed on the same experimental days. Behavioral experiments and statistical analysis were performed by researchers blinded to treatment conditions.

### 2.2. Induction of Osteoarthritis

The coin-flipping method was used to randomize adult and old mice into two groups, saline-treated (controls, CTR) and MIA-treated [28]. Animals were anesthetized with anesthetic isoflurane plus 100% oxygen. Induction of anesthesia was performed in a chamber with 5% isoflurane and maintained with a nose with 0.7% (flow rate = 0.5–1 L/min) [16]. OA was induced by a single IA MIA injection (Sigma-Aldrich, Merck Group, Darmstadt, Germany) into the right knee, 1 mg in a volume of 10 μl of sterile saline [21]. Great caution was used when MIA was injected in the intra-articular space in order to avoid the toxic systemic effect of the compound. CTR mice received an IA injection of sterile saline (10 μL in the right knee).

### 2.3. Morphine Treatment

Control and MIA mice were randomized to either a saline or morphine treatment [28]. Morphine hydrochloride (Salars, Como, Italy) was subcutaneously administered at a dose of 2.5 mg/Kg once daily for 7 days starting from day 7 after MIA injection. This dose was chosen on the basis of previous work from our group performed with other pain models [29], as well as from a preliminary pilot experiment in healthy old animals, where higher doses were accompanied by evident sedation (data not shown). During chronic treatment, all behavioral evaluations were performed before the daily morphine injection. 

### 2.4. Behavioral Testing

Locomotion and hypersensitivity tests were performed before (0, basal) and 3, 7, 10, and 14 days after OA induction. Additionally, the acute effect of morphine treatment on mechanical allodynia was evaluated after the first opioid administration (7 days after OA induction). The frailty index was evaluated before and 14 days after the MIA administration. Before each test, animals were allowed to acclimate to the new environment for 30 minutes. All behavioral assessments were performed in the morning. For pain assessment, three different measurements were recorded for each mouse and the mean was calculated. The means of values obtained from mice of the same experimental group were calculated and used for statistical analysis. 

#### 2.4.1. Weight-Bearing Asymmetry: Incapacitance Test

Changes in the weight bearing on hind limbs were evaluated using the incapacitance tester (Linton Instruments, Norfolk, UK). The instrument is equipped with two independent transduced pads (right and left) capable of measuring, in grams, the load pressure exerted by the hindlimbs of the animal under study. Mice were placed in the Perspex chamber (located above the pads) and maneuvered inside it to stand with one hindlimb on each weighing pad. A reading of weight distribution over the two paws was taken over a 3-sec interval for at least three consecutive measurements [25]. Results were calculated as the weight bearing on the MIA ipsilateral hindlimb as a percentage of the total weight borne by the mouse using the formula: (ipsilateral weight borne) / (ipsilateral + contralateral weight borne) × 100.

A value of 50% represents an equal weight distribution across MIA injected and control hindlimbs, and a value of less than 50% indicates a reduction in the weight bearing on the OA hindlimb. 

#### 2.4.2. Mechanical Allodynia: Von Frey Test

Mechanical allodynia was evaluated using a dynamic plantar aesthesiometer (Ugo Basile, Gemonio, Italy). Mechanical touch sensitivity was assessed using a blunt probe (von Frey filament, 0.5 mm diameter). Briefly, mice were placed in a Plexiglass test cage (w 8.5 x h 8.5 cm) upon a metallic mesh, and the rigid tip of a von Frey filament (punctate stimulus) was applied to the mid-plantar area of the hind paw with increasing force (ranging up to 10 g in 10 s) until the animal felt pain and removed its paw. Responses (Paw Withdrawal Thresholds, PWT) are expressed in grams (g). The test was conducted on both the OA paw (ipsilateral) and the healthy paw (contralateral) [29]. 

#### 2.4.3. Thermal Hyperalgesia: Plantar Test

Thermal hyperalgesia was tested according to the Hargreaves procedure [30], slightly modified by us for mice [31], with a plantar test apparatus (Ugo Basile, Gemonio, Italy). Briefly, mice were placed in Plexiglass boxes (w 11 x h 11 cm) and a constant intensity radiant heat source (beam diameter 0.5 cm and intensity 20 I.R.) was aimed at the mid-plantar area of the hind paw until the animal removed it. Paw withdrawal latency (PWL) was expressed in seconds (sec). The test was conducted on both the OA paw (ipsilateral) and the healthy paw (contralateral).

#### 2.4.4. Locomotor Function: 

Rotarod.

Before the OA induction, all mice were trained on the rotarod apparatus (47650 Rota-Rod NG -Ugo Basile, Gemonio, Italy) for 3 consecutive days. During the training trail, animals learn to balance on a stationary rod (60 sec) and then on a rod constantly rotating at 10 rpm (60 sec). The training procedure was repeated for a total of three trials separated by 10 min intertrial intervals. During test days, two protocols were performed:

- Fixed rotation protocol (10 rpm, cut-off 300 sec) for resistance evaluation; 

- Accelerating protocol (4–20 rpm in 300 sec) for coordination evaluation.

For both protocols, animals were tested three times separated by 15 min inter-trial intervals. The test was completed when the animal fell (time to fall) or the time period ended. Moreover, if during the test, the animal clung to the rod and completed a full passive rotation 3 consecutive times, the timer was stopped, the time annotated, and the test was considered finished [32,33]. For each animal, the results of the three tests, expressed in time, are averaged.

Static rod test.

Coordination and balance were evaluated by assessing the ability of the mice to walk on a 22 mm diameter 60 cm-long round dowel positioned horizontally 80 cm above a padded floor. One rod end, where a closed dark box containing bedding and food was mounted, was fixed to a laboratory bench, and was considered as the arrival. Before the OA induction, mice were trained for 2 days. Briefly, mice were habituated to the arrival box for 3 min and then placed on the rod at increasing distances (10, 20, 30, and 50 cm) and rotated 180° of the arrival box. Once the mouse had learned how to rotate 180° and walk the beam to reach the door without difficulty, the training was considered complete. 

During the test session, the orientation time (t-turn—time taken to orient oneself 180° from the starting position towards the arrival box) and the transit time (t-walk—time taken to reach the arrival box), were recorded for a maximum of 120 s each. The sum of these two parameters represents the total test execution time (t-total). If during the orientation (t-turn) the mouse capsized and/or clung under the pole, fell, or failed to orient itself, both at the t-turn and t-walk, the maximum time was assigned. If during the transit (t-walk) the mouse fell, it was awarded the maximum time [33,34]. Data from three trials were averaged for each mouse.

Grip strength meter.

The grip strength test was used to measure the muscle strength of the combined forelimbs and hindlimbs throughout the Ugo Basile instrument (47200, GSM Grip-Strength Meter, Italy). Briefly, the grasping grid was fitted to a force sensor connected to the peak amplifier, which allowed the automated detection of the animal response (range force detected: 0.1–1500 g). The test was repeated 3 consecutive times for each animal [35] and the values obtained were averaged to obtain the final grip value. 

#### 2.4.5. Frailty Index

The non-invasive 31-item frailty index, based on established clinical signs of deterioration, was performed in adult and old mice before (day 0) and at the end of the experimental protocol (day 14). Briefly, the assessment included an evaluation of the integument, musculoskeletal system, vestibulocochlear/auditory systems, ocular and nasal systems, digestive system, urogenital system, respiratory system, signs of discomfort, body weight (g), and body surface temperature (°C) [23,36]. 

A detailed table with all the parameters evaluated for each system/apparatus is presented in Table S1 in the Supplementary Material. The severity of each deficit was rated with a point scale: 0, no sign of deficit; 0.5, mild deficit; 1, severe deficit. Instead, deficits in body weight and temperature were scored based on deviation from the mean reference value of adult healthy mice according to the following point scale: 0, deviation was <1; 0.25, deviation was 1; 0.5, deviation was 2; 0.75, deviation was 3; 1, deviation was >3. For each mouse, the 31 FI obtained was summed and divided for the maximum possible deficit number (i.e., 31). In this way, a basal FI (day 0) and a final FI (day 14) were obtained. Subsequently, for each mouse, the Δ frailty index (%) was calculated according to the formula: (final FI - basal FI) × 100.

### 2.5. Tissue Collection

A total of 14 days after MIA injection, corresponding to 7 days of morphine treatment, mice were killed by isoflurane overdose. We isolated the spinal cord (L3-L5), ipsilateral DRGs (L3-L5), and ipsilateral sciatic nerve and its distal branches (tibial, common peroneal, and sural nerves) that were snapped frozen in liquid nitrogen and stored at −80 °C. 

Ten mice per group were used to evaluate the mRNA level expressions of interleukin (IL-)1β, IL-6, tumor necrosis factor-alpha (TNFα), glial fibrillary acidic protein (GFAP), cluster of differentiation (CD) 68, and activating transcription factor (ATF) 3 in all tissues. Additionally, in the spinal cord, the CD11b mRNA levels were also detected. Moreover, five mice per group were used to assess (in the spinal cord) the protein levels of IL-6 and TNFα.

### 2.6. RT-qPCR

RNA was extracted from homogenized tissue with TRIzol® reagent (Invitrogen, Carlsbad, USA) according to the manufacturer’s instructions. We used the reverse transcriptase kit LunaScript™ (BioLabs, Ipswich, UK) to obtain cDNA from 1000 ng RNA. Quantitative PCR, by QuantStudio™ 5, using Luna® Universal Probe qPCR Master Mix (BioLabs, Ipswich, UK) was applied, using TaqMan Gene expression assays, (Thermo Fisher Scientific, Waltham, MA, USA) to measure levels of IL-1β, (Mm00434228_m1), IL-6 (Mm00446190_m1), TNF-α (Mm00443258_m1), GFAP (Mm01253033_m1), CD68 (Mm03047343_m1), CD11b (CD11b Mm00434455_m1), and ATF3 (Mm00476033_m1). The mRNA levels were normalized to GAPDH (Mm99999915_g1) and data were analyzed using the 2^(−ΔΔCT) method. Each sample was run in triplicate alongside non-template controls.

### 2.7. Evaluation of Total Protein and ELISA Assay

The spinal cord was homogenized in a lysis buffer (ice-cold phosphate-buffered saline supplemented with 0.05% EDTA and a 0.5% protease inhibitor cocktail (Roche Diagnostics, Monza, Italy) for protein extraction. The samples were then centrifuged at 22.0000 g for 15 min at 4 °C and the supernatant was used to measure both the total protein content (Lowry’s method) and the IL-6 and TNFα cytokines levels by murine ELISA kits according to the manufacturer’s instruction (Invitrogen™, Thermo Fisher Scientific, Waltham, MA, USA). Cytokine concentrations were reported as the pg of cytokine/mg of the total protein content [37].

### 2.8. Statistical Analysis

The statistical analysis was performed using GraphPad Prism 9 (San Diego, CA, USA). Normality and equal variances were checked before choosing statistical tests. Behavioral results were analyzed using two-way repeated-measures ANOVA with Bonferroni’s post hoc test. Data represent the mean ± SD (incapacitance, von Frey and plantar tests) or SEM (rotarod, static rod, and grip strength meter tests) of 15 animals/group. The basal frailty index was analyzed using an unpaired t-test; data represent the mean ± SD of 60 animals/group; the Δ frailty index was analyzed using one-way ANOVA with Bonferroni’s post hoc test, and data represent the mean ± SD of 15 animals/group. Biochemical data were analyzed using one-way ANOVA followed by Bonferroni’s test. Data represent the mean ± SEM of 10 animals/group (for mRNA) or 5 animals/group (for protein). For all analyses, differences were considered significant at *p* ≤ 0.05.

## 3. Results

### 3.1. Hypersensitivity in Adult and Old OA Animals and Morphine Effect

**Figure 1:**
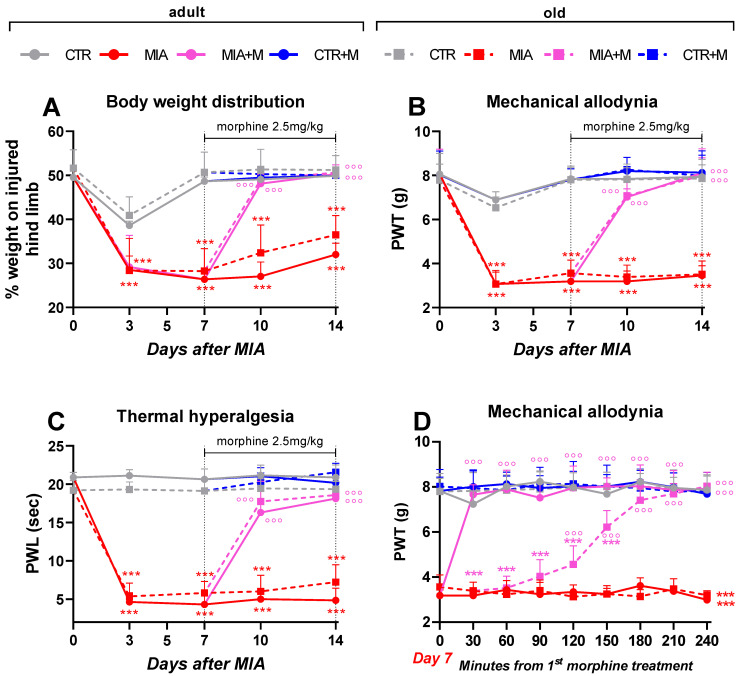
Time course of allodynia and hyperalgesia development. Weight-bearing responses (**A**), mechanical allodynia (**B**), and thermal hyperalgesia (**C**), following MIA intra-articular administration (1 mg in right knee) and morphine treatment (2.5 mg/kg, s.c.) administered once daily for 1-week (from day 7 to day 14) in adult (11-week-old) and old (20-month-old) mice. (**D**) Effect on mechanical allodynia of the first morphine injection (2.5 mg/kg, s.c.) performed on day 7 after OA induction and measured every 30 min up to 240 min. Data represent mean ± SD of 15 mice/group. Statistical analysis was performed by means of two-way ANOVA for repeated measures followed by Bonferroni’s post-test. (**A**) Time × treatments: F (28, 448) = 42.63, *p* < 0.0001; Time: F (3.533, 395.7) = 448.3, *p* < 0.001; treatments F (7, 112) = 200.0; *p* < 0.001. (**B**) Time × treatments: F (28, 448) = 51.23, *p* < 0.001; time: F (3.043, 340.8) = 353.9, *p* < 0.001; treatments: F (7, 112) = 319.1, *p* < 0.001. (**C**) Time × treatments: F (28, 448) = 152.8, *p* < 0.0001; time: F (2.555, 286.2) = 682.9, *p* < 0.001; treatments: F (7, 112) = 463.5; *p* < 0.001. (**D**) Time × treatments: F (56, 896) = 31.73, *p* < 0.001; time: F (6.894, 772.2) = 48.47, *p* < 0.001; treatments: F (7, 112) = 1051, *p* < 0.001. *** *p* < 0.001 vs. respective age-CTR (intra-articular treated with saline), °°° *p* < 0.001 vs. respective age-MIA.

Figure 1 reports the body weight distribution to the limbs (panel A), mechanical allodynia (panel B), and thermal hyperalgesia (panel C) in adult and old mice. Healthy adult and old animals had similar thresholds for the duration of the observational period. The intra-articular injection of MIA elicited a significant weight-bearing deficit (panel A, *p* < 0.001 vs. age-matched controls), mechanical allodynia (panel B, *p* < 0.001), and thermal hyperalgesia (panel C, *p* < 0.001) in the ipsilateral paws of both adult and old animals.

No significant effect of age was detected during the experimental period since adult and old OA animals always displayed similar pain-like behavior. In the contralateral paw, no changes were ever found with respect to the basal thresholds (data not shown). Seven days after the MIA injection, when hypersensitivity was fully developed, animals were daily treated with 2.5 mg/kg of morphine. 

Panel D of Figure 1 reports the effect of acute morphine administration (single bolus) on mechanical allodynia. The treatment significantly and completely reversed allodynia in both adult and old OA animals although with different time courses. In adult animals, the antiallodynic effect was maximal and complete 30 minutes after injection, while in old animals, it steadily increased and was complete 180 minutes after morphine administration. No effect of morphine was observed in healthy adult and old animals. 

One week of daily drug administration was able to fully reverse hypersensitivity in MIA mice, with no age difference (*p* < 0.001 vs. MIA mice of respective age). Morphine did not affect weight bearing, PWL, or PWT in control animals.

### 3.2. Functional Activity in Adult and Old OA Mice and Morphine’s Effect

**Figure 2:**
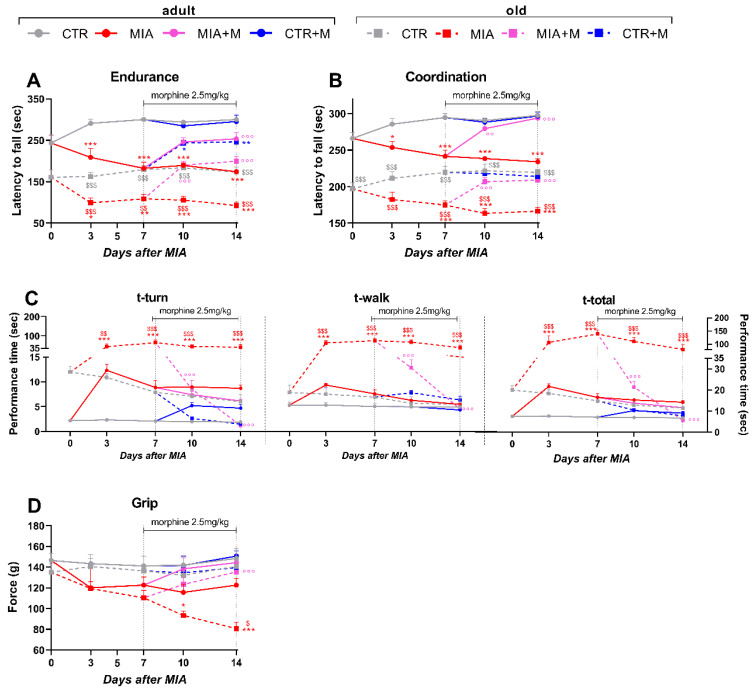
Locomotor activity. Evaluation of locomotor deficits, assessed with rotarod test (**A**), endurance: 10 rpm in 300 s-constant protocol-; (**B**), coordination: 4–20 rpm in 300 s-acceleration protocol-); static rod test (**C**), balance and coordination) and of muscle strength (**D**), assessed by grip strength meter test after intra-articular MIA administration (1 mg in right knee) and morphine treatment (2.5 mg/kg, s.c.) administered once daily for 1 week (from day 7 to day 14) in adult (11 week old) and old (20-month-old) mice. Data represent mean ± SEM of 15 mice/group. Statistical analysis was performed by means of two-way ANOVA for repeated measures followed by Bonferroni’s post-test. (**A**) Time × treatments: F (56, 896) = 31.73, *p* < 0.001; time: F (6.894, 772.2) = 48.47, *p* < 0.001; treatments: F (7, 112) = 1051, *p* < 0.001. (**B**) Time × treatments: F (28, 448) = 3.828, *p* < 0.001; time: F (3.430, 384.2) = 2.819, *p* = 0.0321; treatments: F (7, 112) = 152.3; *p* < 0.001. (**C**) t-turn| time × treatments: F (28, 448) = 4.518, *p* < 0.001; time: F (2.764, 309.6) = 9.967, *p* < 0.001, treatments: F (7, 112) = 23.28, *p* < 0.001; t-walk| time × treatments: F (28, 448) = 8.535, *p* < 0.001; time: F (2.425, 271.6) = 17.85, *p* < 0.001; treatments: F (7, 112) = 36.87, *p* < 0.001; t-total| time × treatments: F (28, 448) = 13.30, *p* < 0.001; time: F (2.516, 281.7) = 29.19, *p* < 0.001; treatments: F (7, 112) = 52.09, *p* < 0.001. (**D**) time × treatments: F (28, 448) = 1.636, *p* = 0.0228; time: F (3.301, 369.8) = 3.896, *p* = 0.0072; treatments: F (7, 112) = 5.773, *p* < 0.001. * *p* < 0.05, ** *p* < 0.01, *** *p* < 0.001 vs. respective age-CTR (intra-articular treated with saline); °° *p* < 0.01, °°° *p* < 0.001 vs. respective age-MIA; $ *p* < 0.05, $$ *p* < 0.01, $$$ *p* < 0.001 vs. respective adult treatment group.

Locomotor activity was first evaluated using the rotarod test. As reported in Figure 2, both endurance (panel A) and motor coordination (panel B) were significantly lower in old mice than in adult ones even in the absence of OA (*p* < 0.001). The presence of OA similarly decreased endurance and coordination in both MIA groups, but considering the lower basal activity of old mice, locomotor activity in these mice was severely affected by OA induction (MIA adult vs. MIA old *p* < 0.01). One week of chronic morphine treatment induced significant improvement of locomotor endurance and coordination in both adult and old OA animals, with values comparable to those registered in the respective CTR mice (MIA vs. MIA + M: adult *p* < 0.001, old *p* < 0.001). We also used the static rod (beam walk test) to assess motor coordination (Figure 2, panel C). Orientation (t-turn), transit (t-walk), and total time (t-total) appeared longer in CTR-old mice than in CTR adult mice, but no statistical differences were present. 

OA induced only a moderate (not significant) increase in the t-turn in MIA adult mice, while orientation (t-turn) and travel (t-walk) times were dramatically impaired by disease in MIA old mice (*p* < 0.001 vs. CTR). Moreover, MIA old animals took much longer than MIA adults to orient themselves (t-turn, *p* < 0.01) and walk the rod (t-walk, *p* < 0.001), resulting in a total time to complete the test (t-total) that was significantly higher (*p* < 0.001). One week of morphine treatment was able to report the performance of old OA mice at the baseline level (*p* < 0.001 vs. MIA old; *p* > 0.05 vs. CTR old).

The four limbs–grip strength test, which measures changes in neuromuscular coordination, muscle intensity, and total functional capability, showed no baseline strength differences between adult and old CTR mice (Figure 2 panel D). The presence of OA induced a reduction in strength in both adult and old MIA mice, but the decline was statistically significant only in MIA-old mice. Indeed, MIA old mice showed a gradual loss of strength compared to CTR old ones, which appeared maximal at the end of the experimental protocol (day 14, *p* < 0.001 vs. CTR old, *p* < 0.05 vs. MIA adult). Moreover, in this case, morphine treatment rescued limb strength in MIA old mice (*p* < 0.001). 

**Figure 3:**
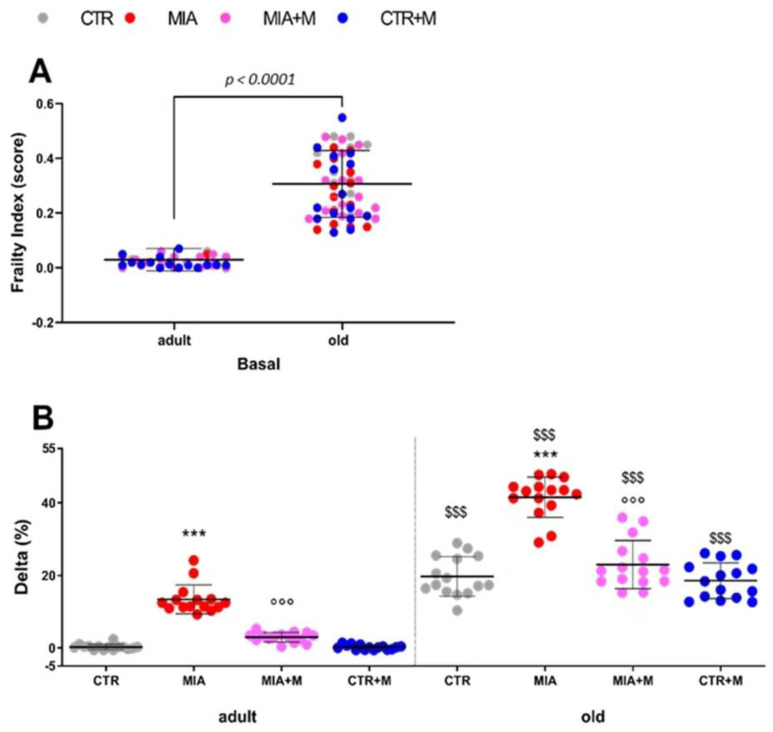
Frailty Index. Frailty index (FI) was assessed at baseline (**A**) and after 14 days from OA. (**A**) Frailty index score of adult (11-week-old) and old (20-month-old) mice before OA induction. (**B**) The delta percentage of basal FI (day 0) and final FI (day 14) of adult and old mice were calculated. OA was induced by intra-articular administration of MIA (day 0; 1 mg in right knee) and morphine treatment (2.5 mg/kg, s.c.) was performed once daily for 1 week (from day 7 to day 14). Data represent mean ± SD of 60 mice/group (**A**) or 15 mice/group (**B**). Statistical analysis was performed by mean of unpaired t-test (**A**) or one-way ANOVA followed by Bonferroni’s post-test (**B**). (**A**) t = 16.68, df = 118; (**B**) Treatments: F (7, 112) = 161.6, *p* < 0.001. *** *p* < 0.001 vs. respective age-CTR (intra-articular treated with saline); °°° *p* < 0.001 vs. respective age-MIA; $$$ *p* < 0.001 vs. respective adult treatment group.

In order to assess the general well-being of the animals and how pain and morphine may affect it, frailty condition was assessed. The frailty index (FI) was evaluated for each mouse at the beginning (day 0) and at the end of the experimental protocol (day 14). Figure 3, panel A, reports the basal frailty indices of all healthy adult and old animals before any treatment. No frailty condition was present in healthy adult animals while aging itself negatively impacted the frailty index, which was significantly higher in healthy old mice than in adult ones (*p* < 0.001). However, it is interesting to note that the presence of OA and related pain significantly worsened the FI in both adult and old MIA mice. Panel B of Figure 3 reports for each mouse the Δ frailty index (%) at the beginning and end of the experimental protocol, calculated as reported in the Method section (*p* < 0.001 vs. respective CTR). Once more, the negative effect of OA was more evident in old than in adult animals, as exemplified by the FI that was significantly higher in MIA old mice than in MIA adult ones (*p* < 0.001). Morphine treatment did not modify the FI in CTR adult and old mice (*p* > 0.05), but it prevented FI from worsening in adult and old OA mice (*p* < 0.001 vs. respective MIA).

### 3.3. Inflammatory Markers in Sciatic Nerve, DRGs, and Spinal Cord of Adult and Old OA Mice and Morphine Effect

**Figure 4:**
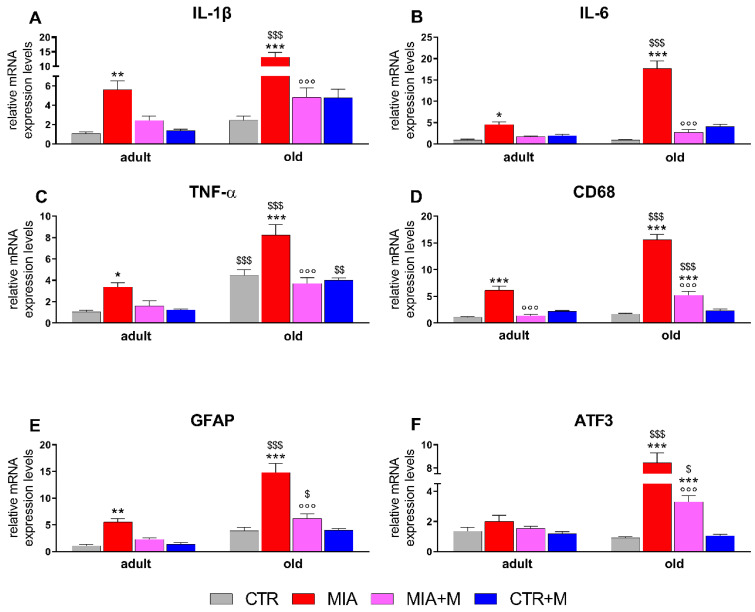
Sciatic nerve. Expression mRNA levels of pro-inflammatory cytokines IL-1β (**A**), IL-6 (**B**), and TNF-α (**C**), macrophage marker CD68 (**D**), Schwann cell marker GFAP (**E**) and cellular damage marker ATF3 (**F**). All the measurements were performed 14 days after OA induction (MIA 1 mg in right knee) and morphine treatment (2.5 mg/kg, s.c.) was performed once daily for 1 week (from day 7 to day 14). mRNA levels, determined by real-time qPCR, are expressed in relation to GAPDH and presented as fold-increases over the levels of CTR adult mice. Data represent mean ± SEM of 10 mice/group. Statistical analysis was performed by means of one-way ANOVA followed by Bonferroni’s post-test. Treatments: F (7, 72): IL-1β= 22.42, *p* < 0.001; IL-6 = 58.09, *p* < 0.001; TNFα = 22.35, *p* < 0.001; CD68 = 81.31, *p* < 0.001; GFAP = 33.15, *p* < 0.001; ATF3 = 46.13, *p* < 0.001. * *p* < 0.05, ** *p* < 0.01, *** *p* < 0.001 vs. respective age-CTR (intra-articular treated with saline); °°° *p* < 0.001 vs. respective age-MIA; $ *p* < 0.05, $$ *p* < 0.01, $$$ *p* < 0.001 vs. respective adult treatment group.

**Figure 5:**
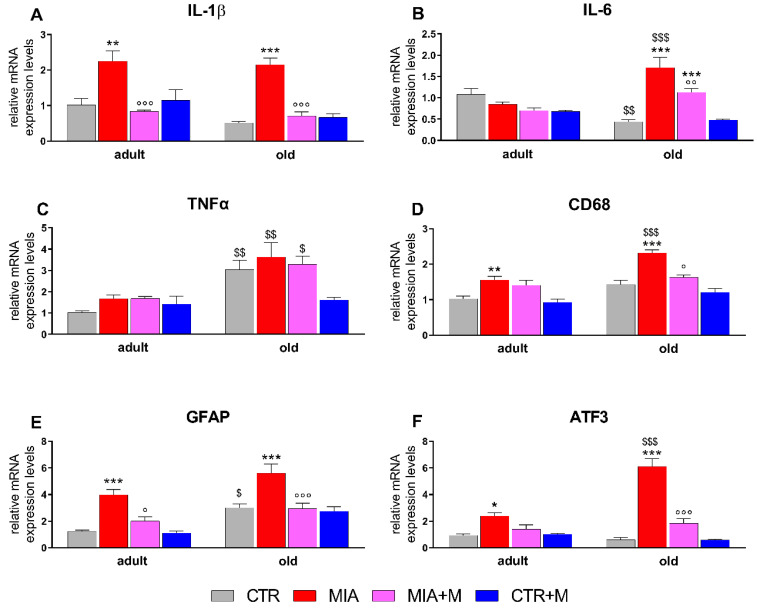
Dorsal root ganglia. Expression mRNA levels of pro-inflammatory cytokines IL-1β (**A**), IL-6 (**B**), and TNF-α (**C**), macrophage marker CD68 (**D**), Schwann cell marker GFAP (**E**) and cellular damage marker ATF3 (**F**). All the measurements were performed 14 days after OA induction (MIA 1 mg in right knee) and morphine treatment (2.5 mg/kg, s.c.) was performed once daily for 1 week (from day 7 to day 14). mRNA levels, determined by real-time qPCR, were expressed in relation to GAPDH and presented as fold-increases over the levels of CTR adult mice. Data represent mean ± SEM of 10 mice/group. Statistical analysis was performed by means of one-way ANOVA followed by Bonferroni’s post-test. Treatments: F (7, 72): IL-1β = 13.76, *p* < 0.001; IL-6 = 16.47, *p* < 0.001; TNFα = 7.99, *p* < 0.001; CD68 = 19.49, *p* < 0.001; GFAP = 15.51, *p* < 0.001; ATF3 = 40.71, *p* < 0.001. * *p* < 0.05, ** *p* < 0.01, *** *p* < 0.001 vs. respective age-CTR (intra-articular treated with saline); ° *p* < 0.05, °° *p* < 0.01, °°° *p* < 0.001 vs. respective age-MIA; $ *p* < 0.05, $$ *p* < 0.01, $$$ *p* < 0.001 vs. respective adult treatment group.

Two weeks after MIA injection, corresponding to 1 week of chronic morphine treatment, proinflammatory cytokines and neuroinflammatory markers were evaluated in the main sites involved in nociception. As reported in Figure 4, in the sciatic nerve of healthy animals, TNFα levels were significantly higher in old mice (*p* < 0.001). In MIA mice, we observed a significant upregulation of IL-1β (adult: *p* < 0.01; old: *p* < 0.001), IL-6 (adult: *p* < 0.05; old: *p* < 0.001), TNFα (adult: *p* < 0.05; old: *p* < 0.001), CD68 (adult and old: *p* < 0.001) and GFAP (adult: *p* < 0.01; old: *p* < 0.001) in both adult and old mice, while ATF3 levels were only significantly increased in older animals (*p* < 0.001). Interestingly, the entity of overexpression was different in the two age groups, being significantly higher in aged animals (*p* < 0.001). Morphine administration blunted the neuroinflammatory markers in OA animals: a significant decrease was seen for adults only for CD68 levels (*p* < 0.001 vs. MIA), while in old mice, it was for all the markers analyzed (*p* < 0.001 vs. MIA). Morphine treatment in CTR adult and old mice did not modify any of the analyzed parameters. In DRGs (Figure 5), TNFα and GFAP expression levels were already higher in CTR-old mice than in adult ones (*p* < 0.01 and *p* < 0.05 respectively, vs. CTR adult). The presence of OA pain increased IL-1β (adult: *p* < 0.01; old: *p* < 0.001), CD68 (adult: *p* < 0.01; old: *p* < 0.001), GFAP (adult and old: *p* < 0.001), and ATF3 (adult: *p* < 0.05; old: *p* < 0.001) in both age groups. As already observed for the sciatic nerve (Figure 4), the levels for most of the neuroinflammatory parameters are higher in old mice (MIA adult vs. MIA old: IL-6 *p* < 0.001; TNFα *p* < 0.01; CD68 *p* < 0.001; ATF3 *p* < 0.001). Moreover, IL-6 overexpression was detected only in MIA-old mice (*p* < 0.001 vs. CTR). The treatment with morphine reduced, when present, cytokine and marker overexpression in old mice (*p* < 0.01 vs. MIA), while in adult mice it resulted in a significant decrease of IL-1β (*p* < 0.001 vs. MIA) and GFAP (*p* < 0.05 vs. MIA) with only a partial reduction of CD68 and ATF3.

**Figure 6:**
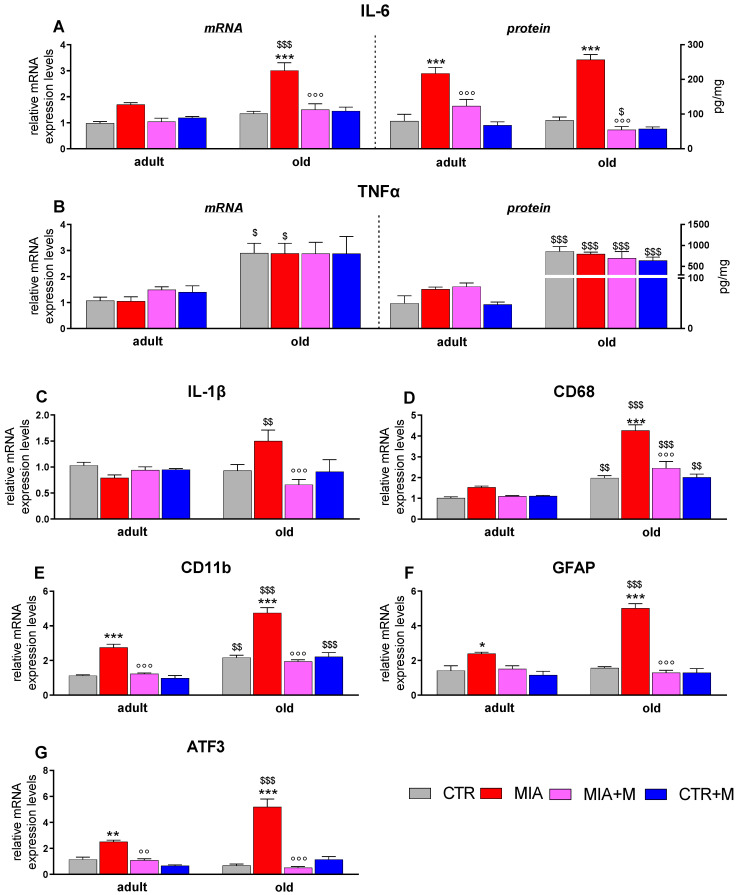
Spinal cord. Expression mRNA and protein level of pro-inflammatory cytokines IL-6 (**A**) and TNF-α (**B**), and mRNA level of pro-inflammatory cytokine IL-1β (panel **C**), macrophage/microglial markers CD68 (**D**) and CD11b (**E**), astrocytic cell marker GFAP (**F**), and cellular damage marker ATF3 (**G**). All the measurements were performed 14 days after OA induction (MIA 1 mg in right knee) and morphine treatment (2.5 mg/kg, s.c.) was performed once daily for 1 week (from day 7 to day 14). mRNA levels, determined by real-time qPCR, were expressed in relation to GAPDH and presented as fold-increases over the levels of CTR adult mice. Data represent mean ± SEM of 10 mice/group. Protein levels were measured by ELISA and expressed as pg of cytokine/mg of total proteins. Data represent mean ± SEM of 5 mice/group. All statistical analysis was performed by means of one-way ANOVA followed by Bonferroni’s post-test. Treatments for mRNA evaluation: F (7, 72): IL-1β = 3.59, *p* < 0.0023; IL-6 = 16.47, *p* < 0.001; TNFα = 6.084, *p* < 0.001; CD68 = 40.95, *p* < 0.001; CD11b = 49.11, *p* < 0.001; GFAP = 40.57, *p* < 0.001; ATF3 = 38.32, *p* < 0.001. Treatments for protein evaluation: F (7, 32): IL-6 = 30.31, *p* < 0.001; TNFα = 23.59, *p* < 0.001. * *p* < 0.05, ** *p* < 0.01, *** *p* < 0.001 vs. respective age-CTR (intra-articular treated with saline); °° *p* < 0.01, °°° *p* < 0.001 vs. respective age-MIA; $ *p* < 0.05, $$ *p* < 0.01, $$$ *p* < 0.001 vs. respective adult treatment group.

We then analyzed the cytokines and the neuroinflammatory markers in the spinal cord, where we also measured CD11b expression. In this tissue, we also evaluated the protein levels of the proinflammatory cytokines IL-6 and TNFα. As reported in Figure 6, also in this tissue, some neuroinflammatory markers, such as TNFα (mRNA *p* < 0.05 and protein *p* < 0.001), CD68 (*p* < 0.01), and CD11b (*p* < 0.01) were already upregulated in CTR old animals in comparison to adult mice, thus indicating a clear microglia activation in healthy old mice. In both adult and old OA mice, significant elevations of IL-6 (adult: mRNA *p* = 0.0545; protein *p* < 0.001; old: mRNA and protein *p* < 0.001), CD11b (adult and old: *p* < 0.001), GFAP (adult: *p* < 0.05; old: *p* < 0.001), and ATF3 (adult: *p* < 0.01; old: *p* < 0.001) were observed, while the increase of CD68 was only present in MIA old animals (*p* < 0.001). Similarly to what was reported for other tissues, the levels of all evaluated mediators were significantly higher in old MIA mice than in adult ones; MIA adult vs. MIA old: IL-6 as mRNA, CD68, CD11b.

GFAP and ATF3 *p* < 0.001; IL-1β *p* < 0.01; TNFα as mRNA *p* < 0.05 and as protein *p* < 0.001). Morphine administration did not modify any marker in CTR adult and old animals, but significantly reduced the overexpression induced by OA pain in MIA adult and old mice (MIA adult vs. MIA + M adult: CD11b, *p* < 0.001; ATF3, *p* < 0.01; MIA old vs. MIA + M old: IL-1β, IL-6, CD68, CD11b, GFAP, and ATF3 *p* < 0.001.

## 4. Discussion

Recently, it was suggested that chronic pain may predispose to frailty in older subjects. Frailty is commonly defined as increased vulnerability to stressors that impair multiple interrelated systems, leading to a decrease in physiological reserves and the ability to maintain homeostasis; pain emerges as one important stressor [23].

Here, we demonstrate that OA of the knee induced similar nocifensive behaviors in adult and old mice, but the presence of pain had a greater impact on motor function, state of well-being, and neuroinflammation in older mice that appeared more vulnerable to the presence of chronic pain. We suggest that prompt and appropriate treatment of pain is successful in mitigating its adverse effects in old mice [15,38].

The neurophysiology of nociception in aging has been studied in recent years, but the data obtained are equivocal, showing either decreased or increased sensitivity to thermal or mechanical stimuli [21,39,40,41]. Differences in species, sex, and type of stimuli may be responsible for the equivocal results. Our results show that basal withdrawal latencies to either mechanical or thermal stimuli were not significantly different between adult and old mice [42]. After MIA injection in the joint, we observed the presence of a weight-bearing deficit and referred pain [42], expressed as thermal hyperalgesia and mechanical allodynia in the ipsilateral paw 3 days after OA induction. We did not observe any age-related differences in the nocifensive behaviors of mice. Only a limited number of studies have evaluated OA hypersensitivity in old animals. In two papers, it was shown that weight-bearing deficits were not different in old and young mice or rats [21,43]. Ogbonna et al. [43] showed how, in 22-month-old mice, mechanical allodynia after MIA injection was milder than in younger animals, but after 4 weeks from OA induction, the differences disappeared. Ro et al. [21] reported that mechanical allodynia is similar in aging, but its duration is longer in old rats. We found that old animals had significantly reduced locomotor activity, motor coordination, and muscle strength already in the basal condition. Although OA pain decreases motor activity in both age groups, the impact of OA pain on physical disabilities in old mice is striking. It is increasingly recognized that in OA patients it is of great importance to enhance physical activity with appropriate therapy besides relieving pain. Our data confirm the importance to evaluate complementary outcomes in animal models, in order to increase clinical relevance [44,45,46,47,48]. Although we are aware that it is difficult to reproduce frailty conditions in mice and elaborate appropriate scales to measure them, the non-invasive–31-item frailty index [23,36] may be a good surrogate [23]. Our results indicate that frailty is not present in healthy adult mice, while it is present in old ones. The presence of pain has a relevant impact on FI in both age groups, indicating that chronic pain may adversely affect life quality during the lifespan, but the well-being of old mice is extremely reduced when pain is present, furthermore indicating an age-related vulnerability. Considering that pain in the elderly has a negative effect on functionality and well-being, we investigated whether an appropriate pain treatment may be beneficial in old mice. Drug treatment in aging subjects is a critical issue since a complex balance between adequate pain treatment without medication overuse and adverse effects is needed to enhance the quality of life and functions [22]. The use of opioid drugs, such as morphine for treating OA pain, is controversial, due to several reported side effects [49,50,51,52]. Studies with the MIA rodent model indicated that morphine efficaciously relieved pain-related behaviors acutely and in a short-term treatment [53,54]. In our study, acute morphine administration significantly reverted nocifensive behaviors in both adult and old mice. Interestingly, we observed a different time course of the analgesic response in the two age groups, since the effect of morphine was significantly delayed in older mice, suggesting a different pharmacokinetics. Daily chronic morphine administration induced a gradual recovery of all pain thresholds. The efficacious relief of chronic pain obtained with morphine also exerts a beneficial effect on locomotor activity and muscle strength, as well as on the frailty index that is reported to the level of the no-pain condition. As far as we know, this is the first evidence of how efficacious pain treatment may affect the frailty conditions in the mouse. 

While the presence of neuroinflammation in the aged brain has been largely confirmed [55], less is known about cytokines and neuroinflammatory markers in the peripheral nervous system and spinal cord during aging. In healthy old mice, we observed that TNFα was higher in all tissue, in agreement with what was reported by other authors [56,57,58], and confirming the existence of an age-associated proinflammatory phenotype. This state is more evident in the spinal cord, where microglial-related markers CD68 and CD11b are upregulated, sustaining the presence of primed microglia in aging [15,59,60]. Our data confirm and expand the concept of the presence of relevant neuroinflammation affecting the sciatic nerve, DRGs, and the spinal cord in the MIA model [18,25,26,38,43]: in the presence of OA and related pain, we observed the development of neuroinflammation in adult animals while the already present neuroinflammatory status was aggravated in old mice. In the nerve, we observed an overexpression of pro-inflammatory cytokines, in agreement with what we previously reported 3 weeks after the MIA administration [20]. As suggested by the overexpression of GFAP and CD68, activated Schwann cells or infiltrating macrophages could be responsible for the increase of inflammatory markers [61]. A similar activation is present also in DRGs, where, as already reported by us and others [20,62] also ATF3 is upregulated. ATF3 is not detectable in intact primary sensory neurons but is upregulated in the nucleus of injured peripheral neurons, such as in rheumatoid arthritis and OA mouse models [24,63,64], suggesting the presence of primary afferent injury. The flow of activation from the nerve to DRGs then proceeds to the spinal cord, where we found relevant microgliosis demonstrated by the overexpression of microglia markers. The increase of GFAP and ATF3 suggests the involvement of astrocyte activation and neuronal damage. In contrast with the established role of microglia in the development of hypersensitivity in the MIA model, the role of astrocytes is less clear, since studies reported either a lack of astrocyte response [25,26] or an increase in GFAP [20,65]. 

In the spinal cord, besides the mRNA levels, we also measured the protein content of the proinflammatory cytokines TNFα and IL-6. The results obtained are superimposable to those found with the evaluation of mRNA, confirming previous studies from our group, performed in other models of chronic pain, where we found good correspondence between mRNA and protein levels [66,67]. Moreover, in the MIA model, other research groups have reported the protein levels of some of the mediators that we present as mRNA, showing similar modulation [9,25,38,68,69,70]. When we compare the neuroinflammatory response in adult and old mice, quantitative differences clearly emerge. In all tissues, and for most considered markers, the upregulation is more evident in OA old mice, confirming the high responsivity of the aged peripheral and central nervous system. Despite the presence of a more sustained neuroinflammatory condition, it is somehow surprising that adult and old mice exert similar nociceptive pain responses after OA induction. The level of activation in the nerve, DRGs, and spinal cord is, however, very significant in the adult mouse with OA, and we can hypothesize that it would be sufficient to overcome a certain degree of activation in order to sustain chronic pain. Our experimental protocol consisted of two weeks of observation; however, since the OA-related hypersensitivity in this model spontaneously decreased starting from 35 days after the MIA administration, it would be interesting to evaluate whether the major inflammatory conditions in the aged mice may correlate with the duration of pain. However, from our results, it clearly emerges that when pain is satisfactorily treated with morphine, the neuroinflammatory status is also significantly blunted, both in adult and old mice, suggesting a relationship between pain and neuroinflammation. Indeed, a one-week treatment with morphine does not affect neuroinflammatory markers in adult and old CTR mice, while it prevented the overexpression of cytokines and neuroinflammation in both OA age groups. On the basis of these observations, the regulatory effect induced by morphine is likely to be ascribed to its efficacious pain-relieving effect rather than a direct modulation of cytokines throughout opioid receptors on glial cells. However, in the literature, chronic morphine administration has been associated with microglia activation and cytokine production in the spinal cord, and this activation was suggested as one of the mechanisms underlying tolerance and opioid-induced hyperalgesia [71,72]. A recent paper reported that 2 weeks of daily administration of morphine in MIA mice did not reverse spinal cord neuroinflammation but rather worsened it [73]. With our morphine dose and time of treatment, we did not observe the development of tolerance to the acute effect of morphine (data not shown). It is possible that the dose and the duration of the morphine treatment may be critical in order to either blunt or trigger microglia activation [73]. We are aware that one limitation of our study is that all evaluations were performed only up to 2 weeks after OA induction. We cannot exclude that a longer period of treatment may elicit different results, but the aim of the study was to perform a prompt and efficacious pain treatment in order to unveil the impact of pain on frailty and prevent it. In old mice with pain, the morphine treatment is only beneficial, since we did not observe adverse effects, as confirmed by the positive effect of the opioid on locomotor activity and frailty index [17,18,26]. We are aware that the length of the treatment used in our study cannot be fully comparable to the duration of chronic pain treatment that produces adverse effects in patients.

In conclusion, we demonstrate that the presence of pain derived from OA, the most common musculoskeletal disease of aging, is a factor that may facilitate the development of a frailty condition during aging. An effective treatment that alleviates pain in the first stage of a painful disease efficaciously reverts functional disability and prevents overt neuroinflammation. From a clinical point of view, although in our study the balance between the risks and benefits of morphine treatment have shifted toward benefits, caution is needed for translating the results to patients.

## Data Availability

Data supporting the findings of this study are available upon reasonable request.

## Supplementary Materials

The following supporting information can be downloaded at: https://www.mdpi.com/article/10.3390/biomedicines10112847/s1, Table S1: Parameters for the frailty evaluation in mice.
