# Supplementary material for: Osteoarthritis Pain in Old Mice Aggravates Neuroinflammation and Frailty: The Positive Effect of Morphine Treatment
Source: Biomedicines. 2022 Nov 8;10(11):2847. doi: 10.3390/biomedicines10112847 (PMC9687902; doi:10.3390/biomedicines10112847)
Supplement: Supplementary file 1 [file biomedicines-10-02847-s001.zip › biomedicines-1944830-supplementary.pdf]

Table S1

Parameters for the frailty evaluation in mice [23, 36].

Integument Apparatus:	
Alopecia	Age-related hair loss (thinning of the hair with hairless areas, especially at the nape of the neck).
Hairs colour loss	Fur changing colour from black to grey or brown.
Dermatitis	Presence of skin erosions or open wounds anywhere on the body, caused by, for example, an excessive grooming or scratching.
Whiskers loss	Loss of whiskers due to aging.
Fur general condition	Messy appearance of the fur, which looks tousled and no longer smooth, sleek and shiny.
Physical/ Muscoloskeletal System:	
Tumours	Development of masses or tumours anywhere on the body.
Distended abdomen	Enlarged abdomen. This may be due to, for example, the growth of a tumour, an organs enlargement or an intraperitoneal fluid accumulation.
Kyphosis	Prominent outward curvature of the spine at the cervical/ lower thoracic level.
Tail stiffening	The tail is stiff and no longer able to wrap around freely when stroked.
Gait disorders	Lack of coordination, including jumping, swinging, and gait.
Tremor	Involuntary tremors at rest or during movement.
Forelimb grip strength	Decrease in the forelimbs grip strength.
Body condition score	Visual signs of muscle wasting or obesity based on the animal size.
Vestibulocochlear/ Auditory Apparatus:	
Vestibular disturbance	Alterations in the ability to perceive motion and gravity, connected with balance, orientation and acceleration problems.
Hearing loss	Failure to respond to a sudden sound (e.g. clicker), indicative of hearing loss or reduction.
Ocular/ Nasal Apparatus:	
Cataracts	Eye lens opacification and opaque spot in the centre of eye.
Corneal opacity	Cloudy cornea with white spots.
Eye discharge/ swelling	Presence of exophthalmia, i.e. swollen and/ or bulging eyes, and may also have abnormal secretions and/ or crusting.
Microphtalmia	Eyes small and/ or sunken, this problem may involve both or just one eye.
Vision loss	Tested by the evaluation of the animal behaviour when it is lowered by the tail and it reaches the ground.
Menace reflex	Evaluation of the presence or absence of a rapid blink and closure of the eyelid fissure in response to a non-tactile visual threat to the eye.
Nasal discharge	Presence of abnormal discharge from the nostrils.
Digestive/ Urogenital Apparatus:	
Malocclusion	Irregular and / or too long incisors.
Rectal prolapse	Protrusion of the rectum from the anal orifice.
Penile prolapse	Impossibility of the penis to re-enter its sheath, indicating the presence of a prolapse.
Diarrhoea	Presence of abnormally shaped feces with litter adhering to it, or feces on the walls of the house cage, or feces, blood or litter around the rectum.
Respiratory System:	
Breathing rate /depth	Development of breath problems such as dyspnoea (difficulty breathing), rales (pulmonary congestion), and/ or tachypnoea (rapid breathing).
Discomfort:	
Mouse Grimace Scale	Measurement of pain / discomfort based on facial expression, assessed through five items: orbital tightening, nose swelling, cheeks swelling, ear position (backward) or moustache change (backward or forward).
Piloerection	Stable condition of involuntary hair erection.
Body temperature:	Body temperature changes relative to an adult reference group.
Body weight:	Body weight changes relative to an adult reference group.

for each item, rating: 0 = absent; 0.5 = mild; 1 = severe.
